# Characterization and Hazard Identification of Respirable Cement and Concrete Dust from Construction Activities

**DOI:** 10.3390/ijerph181910126

**Published:** 2021-09-27

**Authors:** Akshay Gharpure, James William Heim, Randy L. Vander Wal

**Affiliations:** The John and Willie Leone Family Department of Energy and Mineral Engineering and the EMS Energy Institute, Penn State University, University Park, PA 16802, USA; jwh44@psu.edu (J.W.H.II); ruv12@psu.edu (R.L.V.W.)

**Keywords:** cement dust, respiratory hazard, PM_2.5_ characterization, construction, crystalline silica

## Abstract

Construction is an important segment of the economy that employs millions of people. Construction dust is an occupational health hazard to millions of construction workers worldwide. The hazards associated with respirable dust depend upon its particulate size distribution and chemical composition, as these determine the deposition pattern in the respiratory tract and reactivity, respectively. This study presents characterization of the size and composition of the dust from two key construction materials—cast cement and poured concrete. The dust was generated by cutting the cured cement and concrete blocks using an 18” hand-held circular saw as used in highway and building construction. Transmission electron microscopy, scanning electron microscopy, dynamic light scattering, and laser diffraction were performed for the size analysis of the particles. Energy dispersive spectroscopy and X-ray photoelectron spectroscopy were used for chemical analysis. X-ray diffraction was used for phase identification. Electron diffraction patterns were obtained to assess the crystallinity of individual particles. They confirm the crystallinity of particles of different size and shapes. With a particle size range between 0.5 μm and 10 μm, greater than 90% of particles fell below 2.5 μm, presenting a respirable health concern. Crystalline compounds including the metals Al, Ca, Fe, Mg, Na, and K were detected. The concrete particles were most enriched in crystalline silica with a concentration of more than 30% by weight. The presence of metals and high crystalline silica content pose a serious health concern to construction workers.

## 1. Introduction

Cement and concrete are essential elements of modern-day infrastructure. Cement is the most common ingredient used in the construction industry as a binding material which sets and cures over time due to chemical reaction with water. Concrete is a mixture of sand, coarse stones, and cement. Water is added to concrete to activate the cement, which binds the mixture together. By the addition of coarse stones or aggregates, concrete can serve as a building material. Construction activities involving sawing, cutting, and grinding of cast cementitious and cured concrete expose construction workers to a cloud of crystalline dust particles. Exposure to these particles can be a serious health hazard. The Bureau of Labor Statistics estimates that more than 196,000 workers are employed as cement masons and concrete finishers in the USA alone [1]. Their occupation involves smoothening and finishing concrete surfaces with a variety of hand and power tools, which exposes them to the resultant dust. Several other trades including laborers may also perform concrete grinding activities, adding significantly to the total number of exposed workers [2]. In several of these occupations, the crystalline silica exposure from respirable construction dust can exceed by several hundred times that of the NIOSH Recommended Exposure Limit (REL) of 0.05 mg/m^3^ [3]. With such a large portion of the working-age population at stake, there is a necessity to characterize and analyze the dust for its potential adverse health impacts.

In cement plants, cough, sputum, and breathing difficulty is prevalent in exposed workers [4]. Several studies indicate that chronic exposure to cement dust can significantly lower pulmonary functions with duration of exposure [5,6,7,8]. Furthermore, cement dust is also known to cause cancer of the larynx and lung [9,10]. Animal studies have shown that the dust can cause emphysema and fibrosis in lung tissues [11,12].

Cement dust inhalation appears to have secondary impacts as well because of its ability to reach various organs. Pimentel et al. inferred that the inhaled cement particles can enter into the bloodstream and reach the liver because swelling, inflammation, and lesions were found around the liver in cement mill workers [13]. Meo et al. found decreased performance of intercostal muscles and suggested that when cement dust enters the bloodstream, it can also reach and deposit into skeletal muscles, affecting their structure and performance [14]. More recently, inhaled particles were seen to cause disorders in the nervous system [15,16]. Other unhealthy traits such as higher blood pressure and significant increase in weight were found to be statistically prominent in exposed workers [17]. Studies have also revealed an association between cement dust exposure and lowering of hemoglobin and red blood cells in workers [18,19]. Some research groups have also speculated about the translocation of inhaled particles from the respiratory tract to the placenta and fetus, potentially affecting the offspring [20]. Studies have shown that suspended cement particles not only affect cement workers but also residents living near a cement factory or other sources of cement dust from manufacturing operations [21,22,23,24]. 

Differentiated from these workplace and exposure studies are construction activities involving the action of sawing, cutting, or grinding of cementitious materials and concrete. After casting or pouring and subsequent curing, the mineralogic composition of cement and concrete has changed. Well-known mineral forms include portlandite and quartz, each with its own crystalline structure and elemental composition. As often observed at building sites, and sidewalk and highway repair, clouds of dust are generated by the often-used circular saws. The duration of these particles being suspended in air and how far they travel from the source of emission is dependent of the particle size distribution of the dust. The finer particles can remain suspended longer and reach farther distances. In addition, particle translocation and associated secondary impacts seen in various other organs such as liver, heart, spleen, muscles, and so on, are also size dependent, as found in multiple studies [14,15,16,25,26]. Particles finer than 2.5 μm are more hazardous, as they enter deeper into the pulmonary system and bloodstream [27] and further reach other organs. Hence, a study of dust size distribution generated during cutting activities is critical.

Apart from particle size, the chemical composition of the dust also has a direct impact on the hazards posed by the dust particles. With reference to the components of cement and concrete products, crystalline silica is a known abrasive to the lung tissues and the cause of silicosis [28]. Calcium hydroxide causes irritation of the nose and throat with a risk of permanent lung damage [29,30]. Inhalation of dust containing metal content in the dust particles can also contribute to inflammation and lung damage [31]. 

Although the chemical composition of cement and concrete can be easily read off the manufacturer’s data sheet, the chemical phases in the cement undergo substantial changes after hydration and setting. In addition, surface composition can be more important than overall composition, as the particle surface is what will be in direct contact with cell membranes. Surface composition can differ from the overall particle composition, contingent on the way cleavage occurs at the mineral phase boundaries in cement and concrete during the cutting activities. In addition, some of the chemical phases may separate out and preferentially form finer particles, depending on hardness and other physical properties of different phases and dynamics occurring during scission. Therefore, an in-depth study regarding the chemical nature of the dust is crucial for assessing the health concerns.

To understand the risks of cement and concrete dust toxicity, it is necessary to quantify the physical and chemical properties of these particles. Prolonged exposure to even small concentrations of toxins can be a serious health concern. To our knowledge, no study to date has collected and performed size and chemical characterization of actual dust produced by cutting and grinding cement-based construction materials. To date, studies and regulations have focused upon cement dust exposure from factories. However, as routinely observed at construction sites, pre-cast concrete panels require cutting (e.g., for making slots and holes). Similarly, the replacement and repair work observed routinely on highways exposes the road crews (and passers-by) to cement dust during cutting. Workers are commonly seen without respiratory protection; even a mask is not required for such work. Although cutting of cement and concrete is extensive, surprisingly, very little is known about the particulate emissions from such activities. The toxic effects of this inhalation hazard will fundamentally depend upon particulate properties such as size, surface area, chemical composition, and crystalline structure. This study aimed to characterize the physico-chemical properties of cementitious dust as a basis for gauging potential health hazards. Armed with this knowledge, appropriate precautions and protective actions can be implemented. 

## 2. Methodology

**Figure 1:**
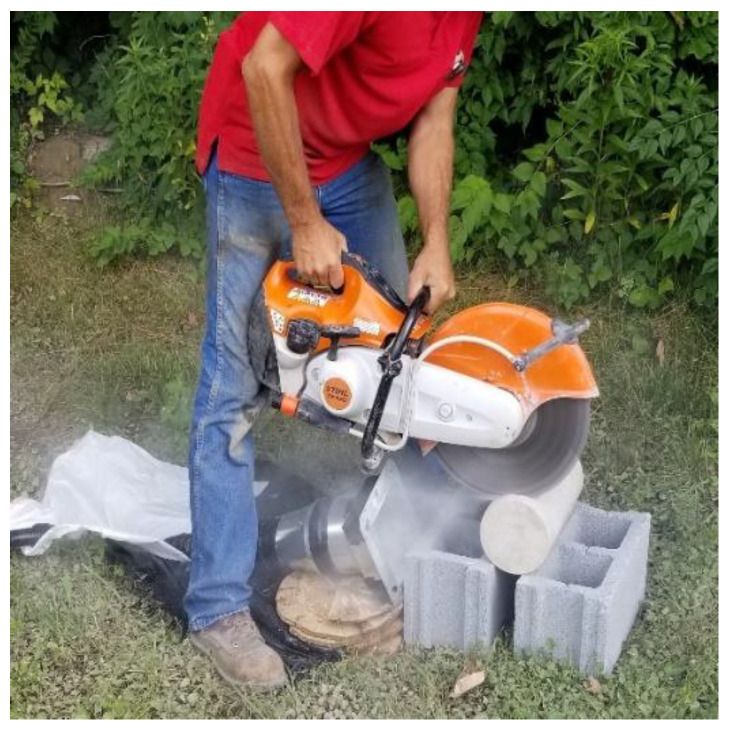
Image showing the dust collection method and apparatus.

The cement paste was prepared by adding water to Portland cement concrete mix (Commercial Grade Quikrete Type I/II), with 67.3% cement by weight and the remainder of water. The mixing was carried out in a clean plastic bucket using a drill connected to a spiral mixing attachment. Cement paste was mixed according to the procedure prescribed in ASTM C305, with the exception that the mixing time was tripled to ensure consistency. The mixture was then poured into the mold. After, the mold was put on a vibrating table for 3–5 min to allow gases to escape. It was then covered for 24 h to set. As shown in Figure 1, the block of set concrete paste was cut using a TS 420 STIHL Cutquik Concrete Saw equipped with a diamond blade, while a custom homemade dust shroud attached to a vacuum collected the generated particles. A block of concrete obtained from a local manufacturer was used to generate the concrete dust using the same saw and collection apparatus.

### 2.1. Laser Diffraction 

Laser diffraction is the most common technique used to determine cement particle size distribution (PSD) [32]. The analysis is based on angular variation in scattered light intensity by particles of varying sizes. The sample solution was prepared by mixing collected cement dust in distilled water (~5% mass fraction) using a magnetic stirrer for 5 min, followed by ultrasonication for another 5 min. A Malvern Mastersizer 3000 was used to carry out laser diffraction measurements. The sample dispersion was added drop-wise into the automated sample dispersion unit containing distilled water with stirring and sonication modes turned on. The solution was added into the dispersion unit until the recommended obscuration (around 7%) was reached. The optimal obscuration eliminates the sampling error and multiple scattering error.

**Figure 2:**
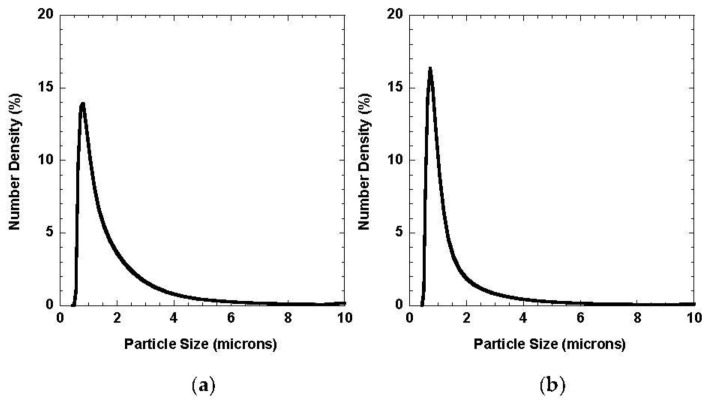
Fits to laser diffraction measurements of (**a**) cement and (**b**) concrete dust.

Cutting with the circular saw blade generates particles with a lognormal particle size distribution (PSD) for both cement and concrete, as seen in Figure 2. It was observed that more than 90% of the particles are less than 2.5 μm in size. The limitation of the laser diffraction measurement is that Fraunhofer and Mie theories are applicable to spherical particles, not the fragmented, irregularly shaped particles predominant in cement dust. Thus, other characterization techniques were employed to study the morphology of the particles.

### 2.2. XRD

**Figure 3:**
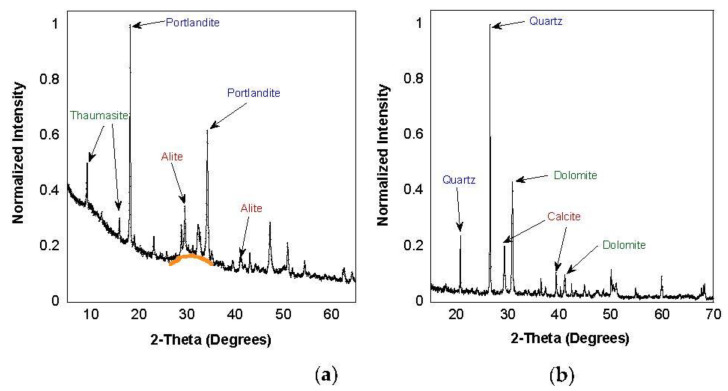
XRD pattern of (**a**) cement and (**b**) concrete dust.

X-ray diffraction is one of the prominent techniques routinely used to identify and quantify crystalline phases in bulk powdered material. The measurements were carried out by a Malvern PANalytical Empyrean diffractometer equipped with a Cu source (λ≅1.54 A°), para-focusing optics, and a PIXcel 3D detector. The spectrum was scanned in the 2θ range of 5° to 90°. Figure 3 shows a portion of the collected spectrum for both cement and concrete dusts. Peaks corresponding to the top three crystalline components in the cement and concrete dust have been highlightedfor illustration. The background subtraction, phase identification, and quantification were performed using MDI JADE^®^ software. Peaks at multiple diffracted angles from different lattice planes were verified for each phase using the database to ensure accuracy in phase identification. The quantification from the fit that yielded the lowest residual is reported in Table 1 and Table 2. 

### 2.3. TEM

**Figure 4:**
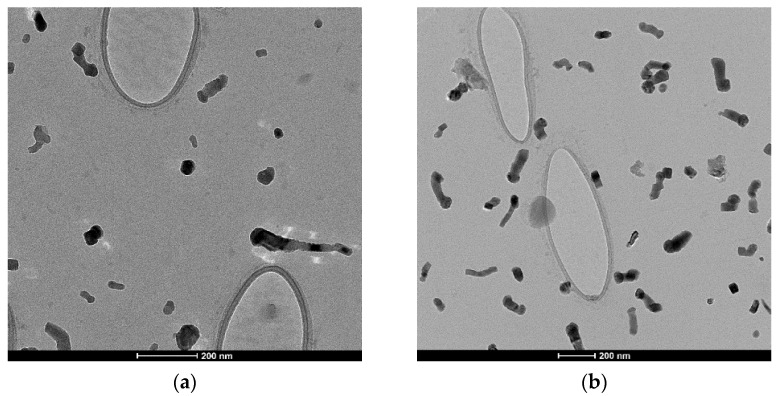
Bright field TEM of (**a**) cement and (**b**) concrete dust on SLG grid.

Transmission electron microscopy (TEM) uses a beam of highly energetic electrons which transmits through the specimen to form an image on a fluorescent screen. The images were taken using a FEI Talos^TM^ F200X scanning/transmission electron microscope equipped with an FEG source providing 0.12 nm resolution. The TEM samples were prepared by sonicating the cement and concrete powder in ethanol for 5 min and then dropping 2–3 drops of the solution on single-layer graphene (SLG) supported on a copper TEM grid. The instrument was operated at 200 kV, and the samples were imaged at various magnifications in the range of 10 kX to 500 kX. Unlike other indirect particle size characterization techniques, direct TEM imaging can also reveal the particle morphology and compositional homogeneity. It was observed that the smaller particles (typically < 50 nm) are spherical, while the bigger particles have highly irregular shapes with sharp edges. Aggregates with varying mass thickness contrast were also observed, indicating inhomogeneous composition of primary particles. TEM images in Figure 4 show that most of the particles are below 100 nm, are non-agglomerated, and are a mix of non-spherical particles.

### 2.4. Electron Diffraction 

**Figure 5:**
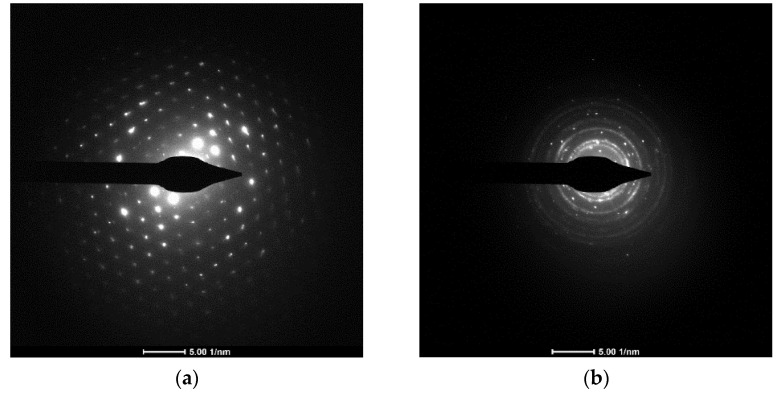
SAED pattern of (**a**) cement and (**b**) concrete dust.

Selected area electron diffraction (SAED) patterns were obtained from samples on lacey carbon to assess the crystallinity of the particles. SAED is a unique technique in that it allows examination of a single particle. Both cement and concrete showed SAED patterns representing highly crystalline particles. A few particles showed a well-defined spot pattern as seen in Figure 5a, representing mono-crystallinity; while others, as seen in Figure 5b, exhibited a mixture of ring and spot patterns, representing both mono-crystalline and poly-crystalline phases in the same particle. This confirms that some elements like copper form separate mono-crystalline particles. The remainder of the particles consisted of a mixture of elements together wherein each element appears in its own crystalline phase.

### 2.5. EDS 

Energy-dispersive spectroscopy (EDS) was performed for elemental composition and mapping in an FEI Talos^TM^ F200X. The EDS mapping was performed in scanning transmission electron microscopy (STEM) mode with a low background sample holder. Using the instrument in STEM mode gives spatial resolution close to the minimum probe size, which is around 1.6 Å. The EDS maps were collected from a sample suspended on a single-layer graphene grid, while the elemental composition data were collected from a sample suspended in the vacuum region of the lacey carbon grid to avoid the background signal from the carbon in the lacy grid.

**Figure 6:**
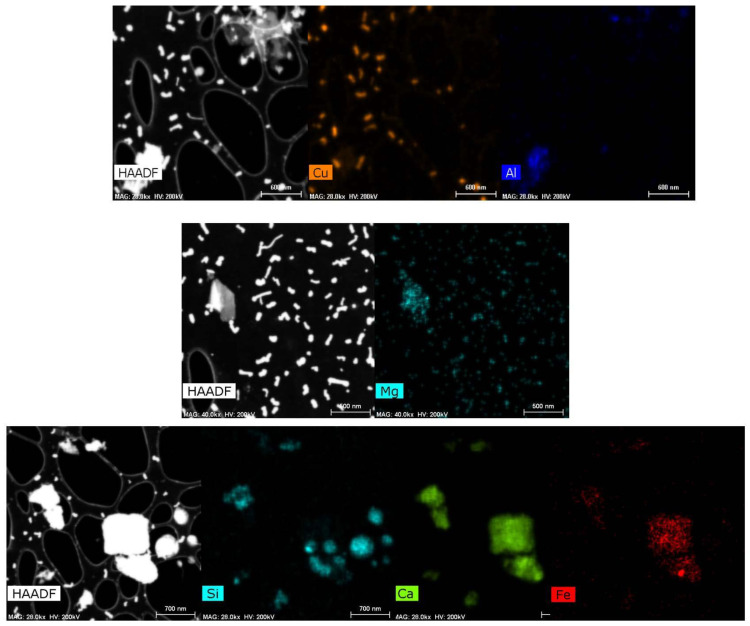
EDS maps showing inhomogeneous elemental distribution in particles.

The EDS maps in Figure 6 show that particles of different size ranges are highly inhomogeneous in their chemical composition. Copper is seen to form a separate phase with relatively smaller particles (<100 nm). Magnesium and iron were found both in small and big particles (>100 nm). Calcium and aluminum were found in large but selective particles, while silicon was found in almost all large particles. Table 3 and Table 4 show the chemical composition of larger size particles (~500 nm).

### 2.6. SEM 

**Figure 7:**
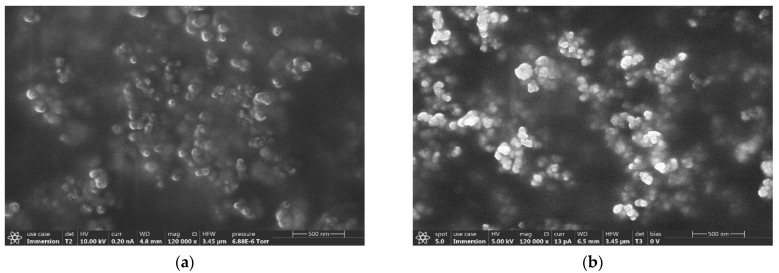
SEM images of (**a**) cement and (**b**) concrete.

**Figure 8:**
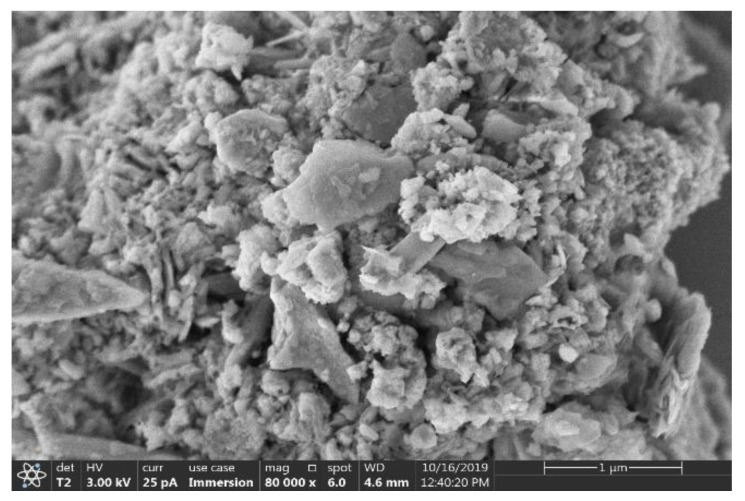
SEM image of representative cement aggregate.

Scanning electron microscopy (SEM) was used to study the surface structure, morphology, and particle size of the samples. The images were taken using Thermo Scientific^TM^ Apreo 2S SEM. The SEM images shown in Figure 7 suggest that the majority of the particles are pseudo-spherical and of size less than 100 nm. The large aggregates have a highly rough surface with many sharp edges and pointed corners. In addition, Figure 8 shows the fine-edged particles seen in SEM have potential for mechanical irritation to the tissues in contact with them.

### 2.7. XPS

**Figure 9:**
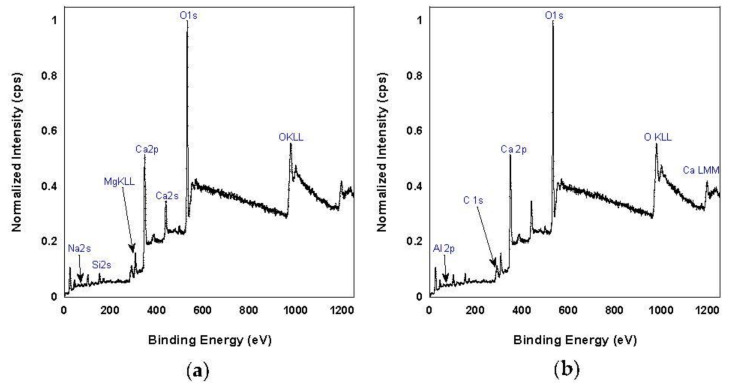
XPS survey spectra of (**a**) cement and (**b**) concrete.

X-ray photoelectron spectroscopy (XPS) is a widely used technique to probe surface composition, typically within the first 10 nm. XPS measurements were performed using a Physical Electronics VersaProbe II instrument equipped with a monochromatic Al kα x-ray source (hν = 1486.7 eV) and a concentric hemispherical analyzer. Charge neutralization was performed using both low-energy electrons (<5 eV) and argon ions. The binding energy axis was calibrated using sputter-cleaned Cu and Au foils. Peaks were charge referenced to the C1s band at 284.8 eV. Measurements were made at a takeoff angle of 45° with respect to the sample surface plane. Figure 9 shows XPS survey spectra from cement and concrete dust. Quantification was done using instrumental relative sensitivity factors (RSFs) that account for the incident X-ray cross section and inelastic mean free path of the emergent electrons. Table 5 and Table 6 give surface elemental composition from quantification of XPS survey spectra of cement and concrete dust.

## 3. Discussion

### 3.1. Particle Size

Particle size is a key characteristic of any respirable dust, as it determines lung penetration and the ultimate fate of the particle thereafter [26]. The particle size distribution due to saw-cutting of cement and concrete was found to be very broad, ranging from ultrafine (<100 nm) to a few microns. This particle size range is similar to what is found in PM emissions from cement plants—0.05 to 10 μm [27]—and hence saw-generated dust from cement and concrete has similar potential for negative health effects as seen in cement plant workers based on particle size.

Most of the primary particles appear roughly spherical. As a measure of physical size, aerodynamic diameter is defined as the diameter of an equivalent spherical particle with unit density and having the same terminal velocity in air as the particle being analyzed. The aerodynamic diameter is thus relevant to particle transport and deposition within the airway passages. The particles between 2.5 and 10 μm are accumulated in the upper part of the respiratory system [27]. The PM_2.5_ (particulate matter < 2.5 μm) can go deeper into the lungs, and some can even enter the bloodstream, and hence pose the greatest health risks [33]. Particles in the range of 7 to 15 μm are deposited in the bronchi and bronchioles [34,35]. Particles larger than 15 μm are generally deposited on the mucous membranes in the nose and pharynx [12].

### 3.2. Composition

Cement can contain various materials which are deemed hazardous, such as calcium oxide (lime), aluminum oxide, magnesium oxide, sulfur dioxide, hexavalent chromium, alkaline oxides, and so on [36]. Silicosis is the most well-known hazard caused by inhalation of respirable dust containing silica and is predominantly an. occupational disease. Crystalline silica is especially hazardous because it is stable, insoluble in water, and generates reactive oxygen species on exposed surfaces which are responsible for oxidative damage to lipids, proteins, and even DNA [37,38]. Silicosis is marked by inflammation and scarring of tissues [39] and is dependent upon severity of exposure. Crystalline silica exposure can also result in many other respiratory diseases such as pulmonary tuberculosis, chronic bronchitis, emphysema, cancer, and other renal and immunologic diseases [38].

Inflammation is believed to be the principal cause for pathogenesis of diseases due to PM exposure [40]. The declining ventilatory function in cement workers is attributed to inflammation [41] and is observed in animals as well as humans subjected to cement dust exposure [42,43]. Meanwhile, the hydration reaction product calcium hydroxide causes irritation of the nose and throat with potential for severe and permanent lung damage [29,30].

As shown here, cement and concrete dust also contains several metal compounds which are of particular concern, as even trace amounts of metals in particulates can generate inflammation via receptor-mediated cell activation or oxidative stress pathways [40]. Reactive oxygen species (ROS), generated by the well-known Fenton reaction (due to iron redox catalysis), are recognized as initiators and mediators of cell death [44]. Inhalation of dust containing magnesium can irritate mucous membranes and the upper respiratory tract [45], while dolomite (calcium magnesium carbonate) causes shortness of breath and reduced respiratory function [31]. Inhalation of aluminum can cause pulmonary fibrosis and lung damage and increase the risk of cardiovascular disease [46,47].

Concrete dust contained more than 30 wt.% of crystalline silica, while cement paste had very little crystalline silica. Most of the silicon in the cement paste was in the form of calcium silicates and in compounds such as thaumasite, lamite, and alite. Cement dust contained more than 35 wt.% of calcium hydroxide in the crystalline phases. Concrete dust contained much higher wt.% of silicon, aluminum, and potassium than cement paste, while the cement was rich in calcium.

## 4. Conclusions

This study reports a comprehensive size and chemical characterization of dust generated during saw-cutting of cement and concrete. Such data is lacking, despite thousands of workers being routinely exposed to this dust. Cutting generates particles of great health concern, as >90% of the particles are smaller than 2.5 μm. Larger aggregates have morphology with sharp edges and protrusions. Concrete dust could have more potential to cause silicosis compared to cement, as it contains >30 wt.% crystalline silica, while cement dust contains mainly calcium silicates. Metal content and other compounds in cement and concrete dust pose a risk of lung damage and other secondary impacts. Most exposure surveys were conducted for the workers exposed to particles from cement plants. No such surveys exist for construction workers performing cement and concrete sawing actions at worksites. Follow-on health effects of these dust particles can be investigated with animal studies. Appropriate precautions and protective equipment should be recommended for the workers being exposed to cement and concrete dust generated during construction activities.

## Figures and Tables

**Table 1:** Quantification of crystalline phases in Cement dust from XRD.

Phases	wt.%
Portlandite	38.5
Thaumasite	31.6
Lamite	7.8
Alite monoclinic	7.3
Calcite	6.9
Gypsum	4.8
Periclase	3.0

**Table 2:** Quantification of crystalline phases in concrete dust from XRD.

Phases	wt.%
Dolomite	45.1
Quartz	32.2
Calcite	18.3
Portlandite	2.2
Pseudo-wollastonite	2.3

**Table 3:** Elemental composition of cement dust from EDS.

Element	wt.%
Silicon	10.10
Calcium	38.32
Oxygen	36.14
Aluminum	1.68
Magnesium	0.30
Iron	1.09
Sulfur	1.73
Carbon	10.64
Chromium	0.008

**Table 4:** Elemental composition of concrete dust from EDS.

Element	wt.%
Silicon	32.56
Calcium	2.79
Oxygen	38.85
Aluminum	9.52
Magnesium	0.31
Iron	0.17
Sulfur	0.08
Carbon	3.20
Potassium	12.40
Sodium	0.132

**Table 5:** Surface elemental composition of cement dust from XPS.

Element	wt.%
Aluminum	1.1
Calcium	17.8
Iron	-
Potassium	0.8
Magnesium	1.2
Sodium	-
Oxygen	59.1
Silicon	4.4
Carbon	15.5

**Table 6:** Surface elemental composition of concrete dust from XPS.

Element	wt.%
Aluminum	3.7
Calcium	12.4
Iron	0.2
Potassium	0.6
Magnesium	4.4
Sodium	2.8
Oxygen	52.7
Silicon	8.8
Carbon	14.3

## Data Availability

Not applicable.
